# Safety and fertility outcomes after the conservative treatment of endometrioid borderline ovarian tumours

**DOI:** 10.1186/s12885-018-5091-1

**Published:** 2018-11-23

**Authors:** Shuang-zheng Jia, Jun-ji Zhang, Zhi-yong Liang, Jun-jun Yang, Yang Xiang, Cong-wei Jia, Jin-hua Leng

**Affiliations:** 1 Department of Obstetrics and Gynecology, Peking Union Medical College Hospital, Chinese Academy of Medical Science & Peking Union Medical College, Beijing, 100730 People’s Republic of China; 2 Department of Pathology, Peking Union Medical College Hospital, Chinese Academy of Medical Science & Peking Union Medical College, Beijing, People’s Republic of China

**Keywords:** Endometrioid borderline ovarian tumor, Fertility, Conservative treatment, Unilateral salpingo-oophorectomy, Cystectomy

## Abstract

**Background:**

Because of the rarity of endometrioid borderline ovarian tumours (EBOTs), there is a paucity of data concerning the natural history and prognosis of this condition. Thus, the objective of our study was to establish the feasibility of fertility preservation in young women with EBOTs, as well as their oncological and reproductive outcomes.

**Methods:**

Consecutive patients with EBOTs, treated at a tertiary referral centre during a span of 22 years, were retrospectively analysed. Recurrence-free interval, as well as its association with the type of surgery and with other clinical and pathological features, was assessed using the Kaplan-Meier and Cox proportional hazards methods.

**Results:**

Of the 59 patients studied, the median follow-up time was 30 months (range, 6–177 months). Nine (15.3%) patients developed 13 recurrences 6–137 months after the initial surgeries, including three patients (5.1%; *n* = 3/59) who developed six invasive recurrences 8, 18 and 68 months after their initial surgeries. Conservative surgery showed a tendency towards a high recurrence rate (17.2% versus 13.3%); however, this difference was not significant (*p* = 0.45). The 5-year recurrence-free survival rate was significantly higher in the oophorectomy group than in the cystectomy group (*p* = 0.001). Cox regression analysis showed that none of the variables assessed were associated with an increased hazard ratio for recurrence, except for a younger age at diagnosis (*p* = 0.021). Of 20 patients who attempted to conceive, three pregnancies among two patients (10.0%) resulted in two live births.

**Conclusions:**

Conservative surgery with unilateral adnexectomy can be proposed for young women with EBOTs with fertility desire; however, the reproductive result is not satisfactory. In addition, careful evaluations of the endometria should be offered during the initial surgery and follow-up period.

**Trial registration:**

Retrospectively registered.

## Background

Borderline ovarian tumours (BOTs) are characterized by features of malignant tumours without destructive stromal invasion and represent 10–20% of all epithelial ovarian tumours [1–3]. Nearly one-third of BOTs are diagnosed in women aged under 40 years old, for whom fertility preservation should be considered [4]. Over the past two decades, conservative surgery that preserve the childbearing potential of young patients is becoming the gold standard for the management of BOTs [1, 5–7]. Evidence from a pooled study showed an estimated cumulative pregnancy rate of 55.7% [8], which increased to 80% after fertility treatment [6]. Even for women with advanced-stage serous BOTs, the pregnancy rate was 57.1% according to long-term follow-up data [9]. However, such conservative surgery is associated with high recurrence rate, although most of which are in the borderline form [8, 10, 11]. Thus, a thorough fertility counselling is urgently needed before proposing conservative surgery [5].

However, data on the safety of fertility preservation in endometrioid BOTs (EBOTs) are lacking, given its low incidence [12–14]. Previous studies on EBOTs have focused mainly on their pathological descriptions [13, 15–17] and included only brief descriptions of the clinical features, management and follow-up of these patients [12, 13, 15]. About one-third of patients with EBOT have synchronous endometrial disorders [13, 15, 17] and concomitant endometriosis [12, 13, 16–18], and half of the patients with EBOTs are nulliparous [14]. However, the fertility outcomes after conservative surgery in these EBOTs patients are not known.

Thus, the objective of our study was to establish the feasibility of fertility preservation in young women with EBOTs, as well as their oncological and reproductive outcomes. To our knowledge, this report is the first to be specifically dedicated to fertility results following the conservative treatment of EBOTs and is the largest series on patients with EBOTs.

## Materials and methods

### Ethics, consent and permissions

As a retrospective study mainly based on medical records, verbal informed consent was obtained from all the patients at their follow-up interviews, and the study was conducted in accordance with the Declaration of Helsinki Principles and regulations of our institute. The Institutional Review Board at the Peking Union Medical College Hospital (PUMCH) approved the study design and this form of consent.

### Study population

Consecutive patients with EBOTs who were treated or referred to our hospital were identified through a search of medical records between 1995 and 2017. Demographic features, disease characteristics, and follow-up records were comprehensively reviewed. Fertility and gestational outcome were completed by telephone interview.

According to the WHO criteria, EBOT was defined as a solid or cystic tumour comprising crowded glands lined by atypical endometrioid-type cells and lacking destructive stromal invasion and/or confluent glandular growth [19]. All pathological reports were examined and confirmed by two expert pathologists at our hospital, and the original pathological slides were re-reviewed by an expert gynaecological pathologist (Z.L.) according to the new WHO criteria [19]. The FIGO 2014 staging system for epithelial ovarian tumours was used to determine disease stage based on the operative descriptions and pathology records [20].

### Surgical management

Conservative surgery was defined as surgery sparing the uterus and some intact ovary to allow further conception. Radical surgery was defined as bilateral salpingo-oophorectomy (BSO) with or without a hysterectomy. A complete staging surgery was considered when all peritoneal surfaces were carefully inspected using peritoneal washing, random or oriented multiple biopsies, omentectomy and removal of any visual tumour [1]. For those patients who were referred to our hospital after the diagnosis of EBOT, a second surgery might have been offered. The extent of surgery was determined by the combination of the previous surgery and surgery performed at our hospital. Data on the use of pelvic lymphadenectomy and adjuvant therapy were also collected.

### Pathological features

Data on specific pathological features, such as microinvasion and intraepithelial carcinoma, were collected. Microinvasion was defined as a BOT with a focus of stromal invasion no more than 10 mm^2^, whereas intraepithelial carcinoma was diagnosed when severe nuclear atypia was observed without stromal invasion [12, 17]. We also retrieved data on coexisting endometriosis (or a previous history of endometriosis) or endometrial disorders, such as endometrial intraepithelial neoplasia (EIN) or endometrial endometrioid carcinoma (EC), during histological analysis or follow-up.

### Statistical analyses

The primary outcome was defined as the recurrence-free interval, which was calculated as the interval of time from the initial surgery to the first recurrence or last follow-up using the Kaplan-Meier method and log-rank test. The associations of clinical, pathological, and surgical variables with the recurrence-free interval were assessed using the Cox proportional hazards methods. Student’s t-test, the Mann–Whitney U-test and the chi-square test were used when appropriate. A *P*-value of ≤0.05 in a two-sided test indicated a significant difference. Statistical analysis was performed using SPSS software (version 25.0; Chicago, IL, USA).

## Results

### Patient characteristics

A total of 980 BOTs were identified during the study period; 59 (6.02%) were diagnosed with EBOTs and were the subjects of this study. The mean age of the patients at diagnosis was 41.7 years (range, 23–81 years). Of 47 women aged ≤50 years at diagnosis, 29 (69.2%) had a conservative procedure, leaving the uterus and some intact ovarian tissue in situ. Of these, 8 patients (20.5%) retained the involved ovary by cystectomy. Compared with the radical group, patients undergoing conservative surgery were younger and more likely to be nulliparous (*p* < 0.001), and the median serum CA-125 and CA-199 values before surgery were lower in the conservative group (*p* = 0.026 and 0.017, respectively).

Complete staging surgery was performed for 32 patients, of which 24 (75.0%) patients had pelvic lymphadenectomy simultaneously. The principal factor appearing to determine the procedure was patients’ age, and only two women under 35 years had pelvic lymphadenectomy. Conversely, laparoscopy was more commonly used in the conservative group (*p* < 0.001). Postoperatively, 17 women (28.8%) received chemotherapy mainly due to advanced stage (FIGO stage IC-IIIC, *n* = 10), intraepithelial carcinoma (*n* = 5), and radiotherapy was prescribed to two women to treat synchronous endometrial cancer.

Histological analysis revealed that 16 patients (27.1%) had associated endometriosis, and seven (11.9%) patients had a history of endometriosis. No significant difference was observed in the endometriosis distribution, intraepithelial carcinoma or stromal microinvasion (*p* = 0.91, 0.14 and 1.00, respectively) between groups. Interestingly, among the 47 patients (79.7%) with endometrial evaluations, a significantly higher number of women were diagnosed with EIN or EC (*p* = 0.026) in the conservative group. The demographic features and disease characteristics of our 59 patients are detailed in Table 1.

**Table 1:** Baseline characteristics of patients with EBOTs

Characteristics	Conservative group (*n* = 29)	Radical group (*n* = 30)	*P* value
Age at first diagnosis (years)	33.9 ± 7.4	49.3 ± 9.9	<0.001^a^
BMI (Kg/m^2^)	22.9 ± 3.7	23.6 ± 2.3	0.45^a^
Nulliparous			<0.001^b^
Yes	23	5	
No	6	25	
Preoperative serum CA 125 (*n* = 54)	50.8 (21.2–327.5)	140.5 (9.5–2655.0)	0.026^c^
Preoperative serum CA 19–9 (*n* = 31)	22.6 (7.5–687.4)	243.3 (10.1–70,148)	0.017^c^
Tumor size (cm)	7.5 ± 3.3	7.5 ± 3.4	0.98^a^
Surgical approach			<0.001^b^
Laparoscopy	21	3	
Laparotomy	8	27	
Complete staging			<0.001^b^
Yes	9	23	
No	20	7	
Stage			0.35^b^
I	28	26	
II-III	1	4	
Microinvasion			1.00^b^
Yes	0	1	
No	29	29	
Intra-epithelial carcinoma			0.14^b^
Yes	9	15	
No	20	15	
Associated endometriosis			0.91^b^
Yes	12	15	
No	17	15	
Endometrial evaluations (*n* = 47)			0.026^b^
Normal	7	18	
EIN/EC	11	11	
Adjuvant therapy			0.18^b^
No	23	19	
Chemotherapy	6	11	

^a^ Student’s *t*-test

^b^ χ^2^ test

^c^ Mann–Whitney *U*-test

*BMI* body mass index, *EIN* endometrial intraepithelial neoplasia, *EC* endometrial endometrioid carcinoma

### Disease outcomes

**Fig. 1:**
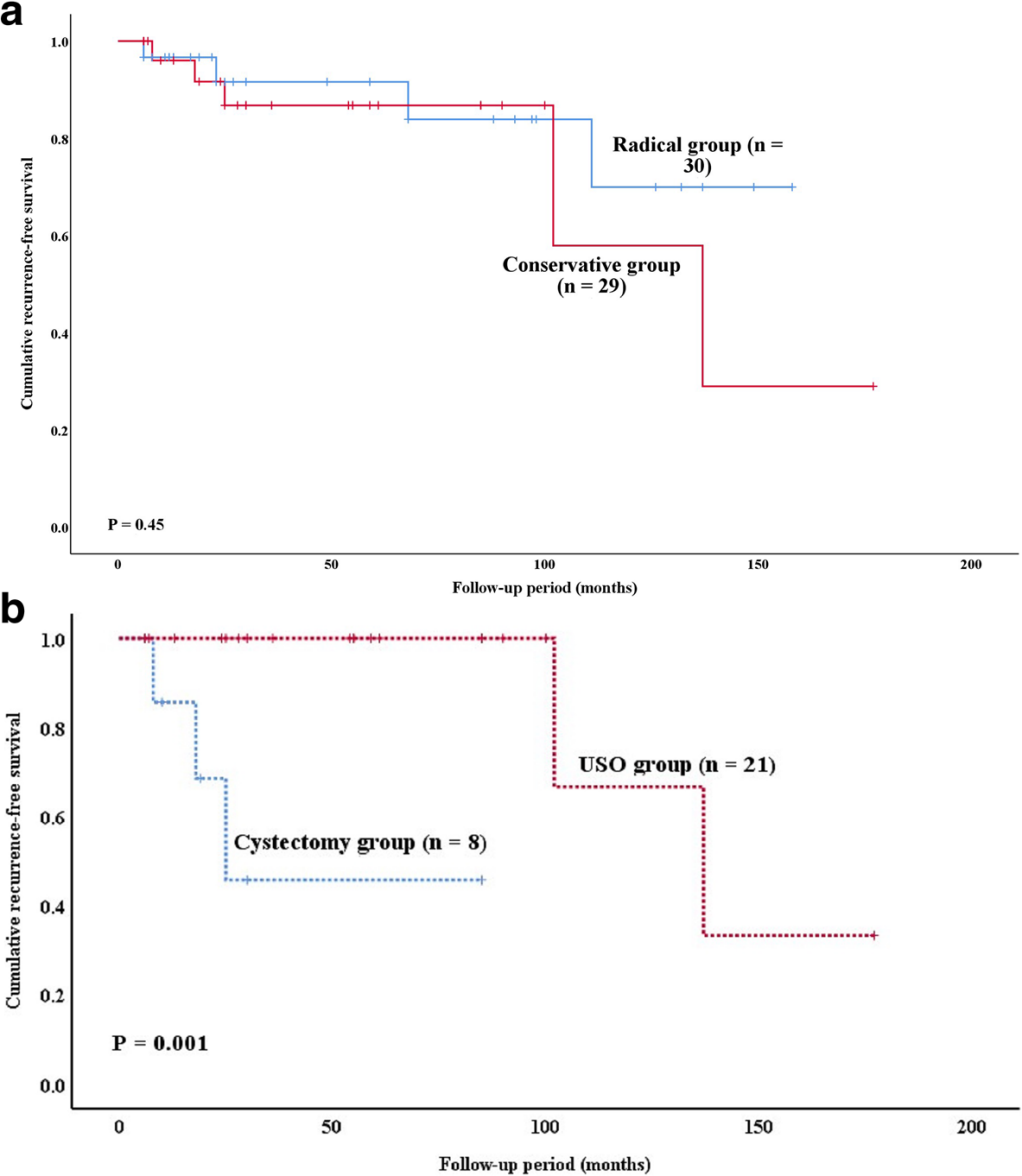
**a** Recurrence-free survival of women with EBOTs (conservative versus radical). **b** Recurrence-free survival of women with EBOTs (UC versus USO)

The median follow-up time was 30 months (range, 6–177 months). Nine patients (15.3%) developed 13 recurrences 6–137 months after the initial surgery (median interval, 25 months), including five patients with seven recurrences in the conservative group and four patients with six recurrences in the radical group. As shown in Fig. 1a, the patients who underwent conservative surgery showed a tendency for earlier recurrence, with a high recurrence rate (17.2% versus 13.3%), but this difference was not significant (*p* = 0.45; Fig. 1a).

Table 2 shows the characteristics of the nine patients with recurrences. The median age of the nine patients with recurrence was 35 years (range, 23–47 years). During the initial management, four patients had complete staging surgery, and five patients received additional platinum-based chemotherapy. All patients had FIGO stage I except for one woman with stage IIIB. In the radical group, the pelvic peritoneum was the most common (75.0%; *n* = 3/4) recurrent site, whereas in the conservative group, the most common site of recurrence was the ipsilateral ovary (60.0%; n = 3/5) that had been preserved at the initial surgery.

**Table 2:** Characteristics of the nine patients with recurrences

No.	Menopausal	CA-125/ CA 19–9	Primary surgery			FIGO stage	Coexisting EMS / ED	Adjuvant therapy	Min. / IEC	RFI (mo)	Recurrence			F/U (mo)	Current status
			Path	Extent	Staging						Sites	Histology	Treatment		
#1	N	217.6 / NA	Lam.	Radical	Yes	IC	Y / N	Chemo.	N / Y	68	Rectum & liver	EOC, twice	RCRS at 1st, chemotherapy at 2nd	80	AWD
#2	N	291.0 / NA	Lam.	Radical	Yes	IC	Y / N	Chemo.	N / N	6	Pelvic	BOT	RCRS	35	NED
#3	N	1037.0 / NA	Lam.	Radical	Yes	IIIB	N / N	Chemo.	N / Y	111	Pelvic	BOT	RCRS	113	NED
#4	N	164.3 / NA	Lam.	Radical	No	IC	Y / N	No	N / Y	23	Contralateral ovary & rectum	EBOT, twice	RCRS at 1st, RCRS and chemotherapy at 2nd	67	NED
#5	N	NA / NA	Lap.	USO	Yes	IC	N / Y	No	N / N	137	Contralateral ovary	EBOT	TAH + USO	147	NED
#6	N	NA / NA	Lam.	USO	No	IC	N / Y	Chemo.	N / N	102	Contralateral ovary	EBOT	Lap. UC	126	NED
#7	N	52.6 / NA	Lam.	UC	No	IC	Y / N	Chemo.	N / N	8	Ipsilateral ovary and LN	EOC, three times	CRS and chemotherapy at 1st, RCRS and chemotherapy at 2nd	48	AWD
#8	N	21.2 / 12.4	Lap.	UC	No	IA	N / N	No	N / N	25	Ipsilateral ovary	EBOT	Lap. USO	31	NED
#9	N	21.7 / 7.5	Lap.	UC	No	IC	N / Y	No	N / N	18	Bilateral ovaries	EOC	Staging	50	NED

*EMS* endometriosis, *ED* endometrial disorders, *Min* microinvasion, *IEC* intraepithelial carcinoma, *RFI* recurrence-free interval, *F/U* follow-up, *NA* not available, *N* no, *Y* yes, *Lam* laparotomy, *Chemo* platinum-based chemotherapy, *EOCs* endometrioid ovarian carcinomas, *RCRS* redebulking surgery, *AWD* alive with disease, *NED* no evidence of disease, *TAH* trans-abdominal hysterectomy, *USO* unilateral salpingo-oophorectomy, *UC* unilateral cystectomy, *LN* lymph node, *Lap* laparoscopy

Notably, three patients (5.1%; n = 3/59) developed six invasive recurrences 8, 18 and 68 months after their initial surgeries. Four recurrences were treated with redebulking surgery plus platinum-based chemotherapy, one with restaging surgery exclusively, and one with three cycles of hepatic arterial infusion of chemotherapy for the second hepatic relapse. All patients were alive at the time of analysis, including two women who survived with tumours.

Kaplan-Meier survival curves for the interval to first disease recurrence are presented in Fig. 1a. As shown in Table 3, none of the variables assessed were associated with an increased hazard ratio for recurrence, except for a younger age at diagnosis (*p* = 0.021). Nevertheless, patients who received conservative surgery or had coexisting endometriosis showed a tendency for recurrence (*p* = 0.059 and 0.071, respectively).

**Table 3:** Hazard ratios for disease recurrence

Variables	Category	HR	95% CI	*P* value
Age at diagnosis (years)		0.86	0.76–0.98	.021
Complete staging	No	1.0		
	Yes	0.35	0.07–1.86	.218
Fertility-sparing surgery	No	1.0		
	Yes	8.64	0.92–80.76	.059
Coexisting endometriosis	No	1		
	Yes	5.15	0.87–30.57	.071
Intraepithelial carcinoma	No	1		
	Yes	0.49	0.07–3.37	.465
Disease stage	I	1		
	II - III	1.06	0.13–8.67	.955

*HR* hazard ratio, *CI* confidence interval

As shown in Fig. 1b, for the women who underwent conservative surgery, the 5-year recurrence-free survival rate was significantly higher in the unilateral salpingo-oophorectomy (USO) group than in the cystectomy group (*p* = 0.001).

### Fertility outcomes

**Fig. 2:**
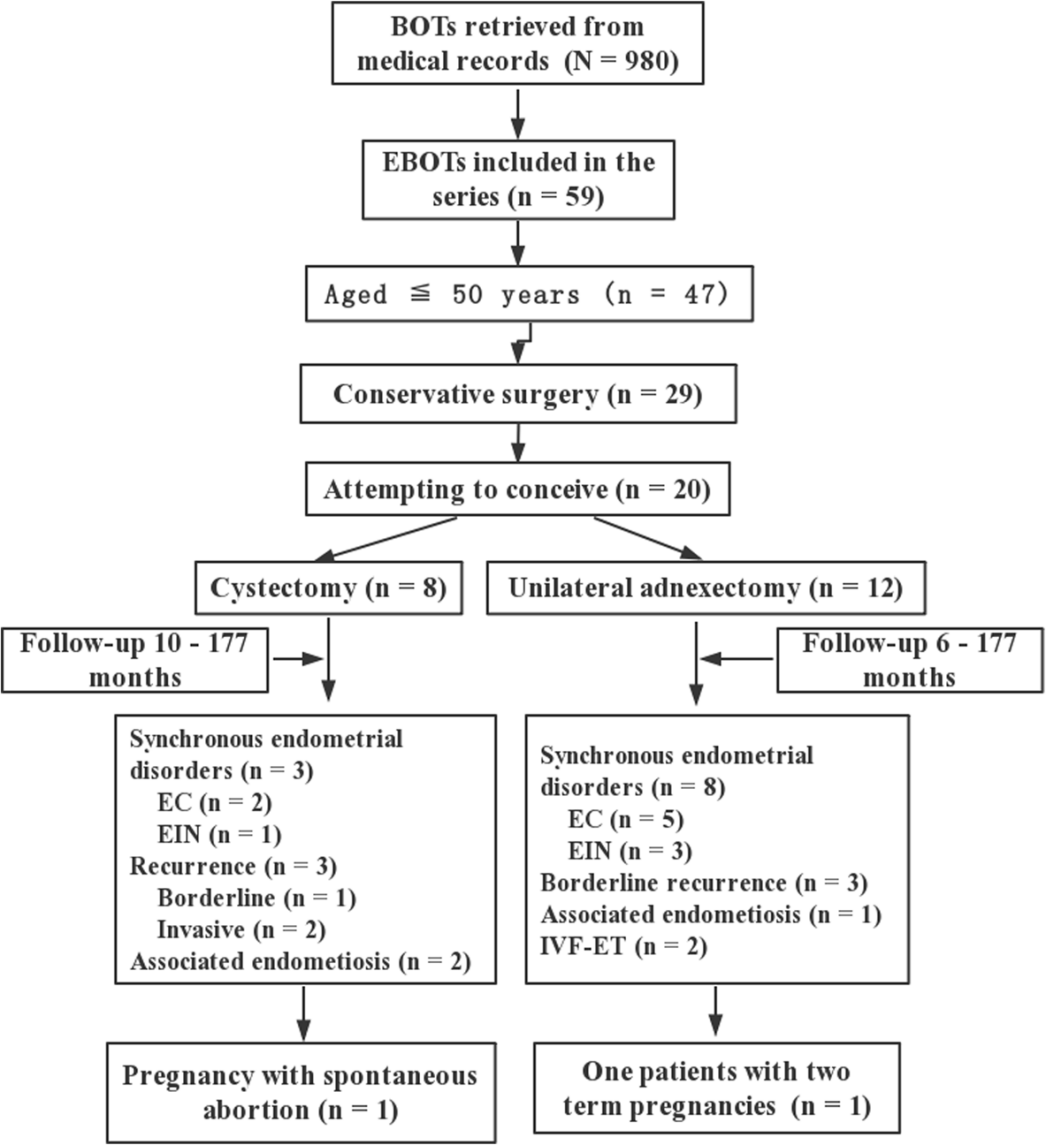
Flowchart of reproductive outcomes underwent conservative surgeries in EBOT patients

Among 47 women aged ≤50 years at diagnosis, 29 (61.7%) underwent conservative procedures, leaving the uterus and some intact ovarian tissue in situ. Of 20 (69.0%) patients who had attempted to conceive at the time of the data extraction, eight (40.0%) received unilateral cystectomies with/without contralateral ovarian biopsies. There were only three pregnancies among two patients (10.0%) that resulted in two live births, even though four women had tried infertility treatments (two with ovarian stimulations and two with IVF-ET). Notably, 11 women developed endometrial disorders (seven with EC and four with EIH) at a median of 18 months of follow-up (range, 0–177 months), and six patients experienced disease recurrence (two invasive and four borderline) at a median of 29 months of follow-up (range, 8–177 months). Details regarding the flowchart of the reproductive outcomes in EBOT patients are provided in Fig. 2.

## Discussion

Conservative surgery is widely accepted as the first-choice treatment for BOT patients with the desire to bear children, with promising oncological and reproductive outcomes. However, because of the rarity of EBOTs, there is a paucity of data concerning the natural history and prognosis of this condition. To our knowledge, this case series is the largest to focus on EBOTs and, specifically, on the feasibility of fertility preservation in young EBOT women. Although restricted by its retrospective nature, this study has several meaningful findings and unanswered questions that need further exploration.

The first interesting result in the current series is the safety of the conservative treatment of EBOTs. Among 29 women who underwent conservative treatment, five experienced seven relapses. This rate of recurrence seemed to be higher than that of their radical counterpart (17.2% versus 13.3%), but this difference was not significant. Consistent with their serous/mucinous BOT counterparts, the ovary that is preserved during the initial surgery is the most common site of recurrence [21–23] and can be salvaged by surgery exclusively in most patients [8, 23, 24]. With respect to EBOTs, similar findings were also reported by Snyder et al. [13] and by Bell & Kurman [16], where five and 13 patients who were treated conservatively had no relapses during a mean of 48 months and 6.3 years of follow-up, respectively. Recently, Uzan et al. [12] also reported seven EBOTS with conservative management (5 with USO alone and 2 with unilateral cystectomy), and one invasive recurrence was observed on the same side of the initial adnexectomy. Consequently, in combination with our present series, conservative surgery that preserves fertility potential can be proposed to young EBOT patients with careful follow-up.

Defining the risk factors of recurrence is crucial towards identifying patients who are at high risk. In the current series, we demonstrated that a young age at diagnosis was the only prognostic factor for recurrence. These results are in agreement with those of Uzan et al [25], who reported that a young age (< 30 years) was the only independent prognostic factor for SBOT recurrence after conservative surgery. Similarly, in a retrospective multicentre study including 950 patients with BOTs, Trillsch et al reported that recurrence was significantly more frequent in patients < 40 years old (19.0% versus 10.1% 5-year recurrence rate, *p* < 0.001) [26]. Furthermore, a retrospective analysis of a French multicentre prospective database demonstrated that BOT patients with recurrence were 11 years younger than those who were recurrence free (32.24 years versus 43.83 years, *p* = 0.0009) [27]. In fact, as demonstrated in our study, patients at a younger age are more likely to receive conservative surgery, which is significantly associated with recurrence risk [10, 28]. In this series, conservative surgery showed a trend towards relapse, although the trend was not significant (*p* = 0.059), which might be due to the small sample size. In contrast to their endometrioid epithelial ovarian cancer counterparts [29], in EBOT patients, coexisting endometriosis seemed to be associated with a high recurrence risk (*p* = 0.071). Thus, combined with a younger age, the role of the hormonal “field effect” in the development of EBOTs needs further exploration. With respect to the traditionally studied prognostic factors [8, 10, 28, 30], neither advanced stage nor complete staging was found to be associated with the risk of recurrence. Similarly, in the classical systematic review by Uzan et al [12], the authors found that all but three EBOT cases were stage I disease and suggested that the peritoneal stage could be omitted in obvious early-stage EBOT.

When cystectomy is compared with USO, the finding that ultraconservative treatment (a cystectomy) increases the risk of recurrence is unsurprising. As demonstrated in serous/mucinous BOTs, ultraconservative surgery is an independent prognostic factor for disease recurrence [8, 10, 11], and unilateral adnexectomy is advisable in cases of mucinous BOTs [8, 11]. In contrast, in women with bilateral BOTs, bilateral cystectomy has been suggested as this method increases reproductive outcomes without increasing the recurrence rate compared to USO plus contralateral cystectomy [31]. Fortunately, an EBOT is mainly a unilateral tumour. To date, only five bilateral cases have been reported among 98 EBOT patients [12, 13, 15, 16]. In addition, in the present series, five bilateral tumours were found. Thus, regarding conservative surgery in this context, unilateral adnexectomy should be proposed rather than cystectomy [12] in patients with EBOTs. However, for the uncommon bilateral cases, the preservation of a maximally healthy ovary should be discussed before further studies are performed with a larger sample size to provide a definitive conclusion [25].

Notably, three cases (5.08%) developed six invasive relapses with a median of 18 months for their first recurrences in our series, which seemed higher than the 2.3% incidence of malignant transformations reported in a large multicentre study that included 950 BOTs [10]. However, whether such invasive relapses were due to fertility-sparing surgery or to the natural history of the tumour was indeterminate. Of our six invasive recurrences, one occurred in the conservative group, and five (two patients) occurred in the radical group. Additionally, as reported by Uzan et al., a 37-year-old woman had suffered an invasive relapse of ovarian endometrioid carcinoma 7 months after the radical treatment of her first borderline recurrence [12]. Thus, together with our current series, radical therapy that sacrifices the uterus and both ovaries does not seem to reduce the risk of invasive recurrence, even with complete radical staging.

Recently, several pathological features, such as invasive peritoneal implants, micropapillary patterns, advanced stage, and the stromal microinvasion, have been proposed to define “high-risk” SBOTs in which disease is likely to evolve into invasive disease [6, 7, 24, 30, 32–34]. For MBOTs, intraepithelial carcinoma and the use of cystectomy seems to be associated with invasive relapse [11, 35, 36]. However, for EBOTs, no such data are available. Among our 59 cases, 24 had intraepithelial carcinomas, whereas only one had an aggressive relapse. Meanwhile, none of the three lethal recurrence cases had stromal microinvasion. Similar findings were also reported by Uzan et al. [12], and a molecular study has demonstrated that EBOTs harbour both KRAS and PIK3CA oncogene mutations [37]. Thus, with such limited data, these pathological features seem to have no effect on overall survival and do not influence the type of surgical management. Furthermore, as demonstrated by du Bois et al., most invasive relapses occur during the first 2 years [10], implying that an intensive post-operative follow-up is necessary.

Concerning fertility results, accumulative evidence suggests the use of fertility preservation in BOT patients with fertility desire [6]. In addition, even for women with advanced-stage serous BOTs, favourable prognoses and satisfying reproductive outcomes have been demonstrated [9, 21]. However, among 20 EBOT patients who tried to conceive in the current series, only three pregnancies between two women were achieved. This rate is significantly lower than that of the serous/mucinous counterpart, and even patients with early-stage epithelial ovarian cancers treated with fertility-sparing surgery conceived at a rate of 60–80% [38, 39]. Notably, 70% of our 20 patients developed EIN/EC during their initial surgery or follow-up periods (Fig. 2), resulting in their need to compromise the uterus and their fertility potential. Previous studies have demonstrated that 20–50% of EBOT patients have synchronous endometrial disorders, and a literature review has shown that among 111 EBOT patients with endometrial evaluations, 41.4% developed synchronous endometrial disorders [14]. Additionally, coexisting endometriosis was found in 40% (*n* = 8/20) of the study patients and would also sacrifice their fecundity. Similar findings were also reported by Roth et al. [17]; among 30 cases of EBOTs, 67% has associated endometriosis. In addition, in the series by Bell & Kurman, of 33 EBOT cases, 12 had endometriosis [16]. Given the high prevalence of coexisting endometriosis and endometrial disorders among the EBOT cases, and all 28 patients evaluated for steroid hormone status of EBOT were positive in the immunohistochemical analysis (unpublished data). Thus, whether hormone therapy could be offered to these women for long-term management is an issue awaiting further study. In addition, adherences and alterations in ovarian reserve after surgery might also contribute to these poor fertility outcomes.

The ultimate aim of fertility-sparing surgery in patients with BOT is to fulfil their childbearing desire, and it’s increasingly recommended that young BOT patients should accept oncofertility counselling prior surgery [5]. Recently, techniques in assisted reproductive medicine have advanced, with treatments ranging from routinely utilized embryo cryopreservation to oocyte and ovarian-tissue cryopreservation, which help retain the possibility of pregnancy without impairing oncological outcomes [40]. In addition, promising results have been demonstrated in terms of the safety and effectiveness of reproductive medical treatments after fertility-sparing treatment for gynaecological cancers [6, 40]. Additionally, in vitro data have suggested that gonadotrophins and/or high-dose oestrogens do not stimulate cultured BOT cell proliferation [41]. In our current study, only one patient received two cycles of IVF-ET treatment, but the treatments failed. This patient had undergone a right laparoscopic adnexectomy and peritoneal biopsies for EBOT at 24 years of age. Twenty months later, she had developed endometrial hyperplasia without atypia, which was successfully reversed with progestin treatment. Afterward, she underwent two cycles of IVF-ET treatment but failed to achieve an ongoing pregnancy after embryo transfer. Approximately 11 years after her initial surgery, a hysterectomy plus left salpingo-oophorectomy was performed due to a borderline recurrence on her left side. She is currently disease-free 14 months after the second surgery. However, the two patients who eventually became pregnant did not receive any assisted reproductive treatments. Given the small sample size, detailed counselling on potential risks and effects should be provided to patients before IVF-ET is offered. Thus, an extensive oncofertility counselling should be proposed to all young women with BOTs, and fertility counselling should become an integral part of the clinical management of women with BOTs [5].

## Conclusions

In conclusion, our series demonstrates that conservative management can be proposed in young women with EBOTs to preserve their fertility and obtain spontaneous pregnancies, but patients should be warned about the high risk of recurrence if an ultraconservative option is used. Given the high prevalence of synchronous endometrial disorders, careful evaluations of the endometria should be offered during the initial surgery and follow-up period. Moreover, the reproductive result is not satisfactory, and an exhaustive oncofertility counselling is advocated. An assisted reproductive procedure can be proposed with caution.

## Abbreviations

BOTs: Borderline ovarian tumours
BSO: bilateral salpingo-oophorectomy
EBOT: Endometrioid borderline ovarian tumors
EC: endometrial endometrioid carcinoma
EIN: endometrial intraepithelial neoplasia
USO: unilateral salpingo-oophorectomy
